# Metal-Organic Frameworks for Wastewater Decontamination: Discovering Intellectual Structure and Research Trends

**DOI:** 10.3390/ma15145053

**Published:** 2022-07-20

**Authors:** Muhammad Nihal Naseer, Juhana Jaafar, Hazlina Junoh, Asad A. Zaidi, Mahesh Kumar, Ali Alqahtany, Rehan Jamil, Saleh H. Alyami, Naief A. Aldossary

**Affiliations:** 1 Department of Engineering Sciences, Pakistan Navy Engineering College, National University of Sciences and Technology, Karachi 75300, Pakistan; 2 Advanced Membrane Technology Research Centre, School of Chemical & Energy Engineering, Universiti Teknologi Malaysia, Skudai Johor 81310, Malaysia; juhana@petroleum.utm.my (J.J.); hazlina.junoh@gmail.com (H.J.); 3 Department of Mechanical Engineering, Faculty of Engineering Science and Technology, Hamdard University, Karachi 74600, Pakistan; asad.zaidi@hamdard.edu.pk (A.A.Z.); mahesh.kumar@hamdard.edu.pk (M.K.); 4 Department of Urban and Regional Planning, College of Architecture and Planning, Imam Abdulrahman Bin Faisal University, Dammam 31441, Saudi Arabia; amalqahtany@iau.edu.sa; 5 Department of Building Engineering, College of Architecture & Planning, Imam Abdulrahman Bin Faisal University, Dammam 31441, Saudi Arabia; rjamil@iau.edu.sa; 6 Department of Civil Engineering, College of Engineering, Najran University, Najran 55461, Saudi Arabia; shalsalem@nu.edu.sa; 7 Department of Architecture, Faculty of Engineering, Al-Baha University, Al-Baha 65528, Saudi Arabia; dr_naief@bu.edu.sa

**Keywords:** metal-organic framework, MOF, wastewater, water treatment, bibliometric analysis

## Abstract

Due to their simple synthesis method and excellent properties, such as superior adsorption and regeneration capabilities, with a large surface area and tunable pores, metal-organic frameworks (MOFs) have emerged as a suitable option for wastewater treatment. Although an exponential growth in MOF literature has been observed in recent years, conducting a quantitative literature analysis of MOF application in wastewater treatment is a novelty. To fill this gap, a total of 1187 relevant publications were extracted from the Web of Science, published during the last 50 years, and analyzed using bibliometric and content analysis techniques. A bibliometric analysis was conducted to reveal growing publication trends, leading journals, prolific countries, and organizations; whereas, a content analysis was used to highlight key research themes and hot topics in this field. The analyses revealed that there is a strong international collaboration among authors, countries, and organizations. *Chemical Engineering Journal*, *Journal of Hazardous Materials*, and *Journal of Environmental Chemical Engineering* are the most prolific journals in this field. Furthermore, the use of MOFs for removing antibiotics from wastewater was identified as a recent hot topic. In addition, performance enhancements of MOFs, in terms of a higher adsorption capacity and water stability, were identified as topics of great interest. To cater to these issues, the application of graphene, graphene oxides, nanoparticles, and quantum dots was also observed in the research fronts in this field.

## 1. Introduction

Issues regarding water resources are mostly faced in developing countries, since it is quite challenging to deliver good quality water for both drinking and household applications. This problematic issue is mostly caused by uncontrollable climate changes and an enormous increase in population, with related anthropogenic activities [1]. A possible option to meet the growing water demand is to reuse wastewater after treatment. Wastewater can be divided into three categories: domestic wastewater, industrial wastewater, and storm wastewater [2]. Notably, domestic wastewater results from the water used in residences, businesses, and restaurants; whereas, industrial wastewater is discharged from the manufacturing and chemical industries. Storm wastewater mostly reflects the contamination of surface runoff water, which picks up the debris, grit, nutrients, and other chemicals in urban and agricultural areas. 

These untreated wastewaters can contain many biological, chemical, and physical contaminants. For instance, the biological contaminants may consist of bacteria (salmonella, campylobacter, shigella), viruses (rotavirus, hepatitis, enterovirus), protozoa, and helminths (roundworm, hookworm, and whipworm), as well as non-pathogenic organisms such as arthropods, insects, and fish. The chemical and physical contamination mainly consists of heavy metals, organic particles, soluble organic materials, macro-solids, emulsions, and toxins. Thus, there is a dire need to devise wastewater treatment methods that can help us to utilize wastewater and, ultimately, pave the way to ensuring good quality water for future generations. The main goal of the treatment is to reduce or remove pollutants, including disease-causing organisms [3]. There are various wastewater treatment methods available in the literature, each of which has its own merits and demerits, as discussed in previous studies [4,5,6,7]. 

From the literature, it is evident that photocatalytic oxidation, adsorption, separation processing, coagulation-flocculation, precipitation, and bioremediation are the most common technologies used for wastewater treatment [8,9]. Some of these techniques are inefficient, have operational difficulties, require large amounts of energy, and are not economically advantageous compared to adsorption techniques. However, the use of nano-based materials to deal with these shortcomings and enhance the efficiency of these processes has recently increased. Nanoparticles such as titanium dioxide (TiO_2_), carbon nanotubes (CNTs), silver (Ag), tin oxide (SnO_2_), zinc oxide (ZnO), gold particles, activated carbon, and graphene have shown improvements, in terms of their cost-effectiveness and efficiency. However, these materials suffer from complicated synthesis procedures [8,9], which limit their potential use for wastewater treatment in the industrial sector. 

To cope with this complex synthesis of nanomaterials, a new class of porous materials was introduced. Metal-organic frameworks (MOFs) are a new class of porous crystalline solids assembled from single metal ions or their clusters, coordinated by organic linkers with a strong bond [10,11,12]. The organic linkers are generally mono-, di-, tri-, or tetravalent ligands. The combination of the type of metal along with the linker defines the characteristics and properties of a MOF. Due to their extraordinary, yet controllable, structure MOFs have been broadly studied for different applications, such as gas storage [13], sensors [14], separation medium [15], catalysis [16], and biomedical applications [17]. The application of MOFs in wastewater treatment is not an exception. Despite a simple and straightforward synthesis method, MOF possess superior adsorption and regeneration capabilities, with large surfaces and tunable pores at low temperatures, which are suitable for the wastewater treatment process [18,19,20]. In addition, the convertible properties of organic linkers, by altering the geometry, length, ratio, and functional group of MOFs, make them potential candidates for targeted pollutant removal from wastewater [8,19,20,21].

In the past few years, MOFs have shown remarkable performance in wastewater treatment, due to their flexible structure. Notably, water stability is an important factor for MOF wastewater treatment applications [22]. Thus, water-stable MOFs are commonly developed for wastewater treatment processes, since such MOFs can maintain their structure in water conditions. The first breakthrough in MOFs for wastewater treatment was in 1997 by Yaghi et al. [23], when it was observed that the performance of zeolites and activated carbon (AC) was deteriorated due to structural defects; thus, the need for water-stable materials became a necessity [18]. 

In the absence of a commonly accepted nomenclature for these materials, such as MOF, in the early discoveries, zeolite-like MOFs, porous coordinated networks (PCNs), microporous coordinated polymer (MCP), and isoreticular MOFs were the names assigned to these materials [18]. Soon, MOFs became popularized and various researchers introduced different MOFs. Particularly, MOF-5, MOF-74, PCN-222, the Material of Institute Lavoisier MIL-53, University of Oslo UiO-66, and Zeolitic Imidazolate Frameworks (ZIFs) have received much attention for wastewater treatment [18,24]. Some other MOFs used for wastewater treatment are listed in Table 1. 

In recent years, MOFs have received much attention as potential candidates for wastewater treatment. However, conducting a quantitative literature analysis of MOF applications in wastewater treatment represents a novelty. Although a few studies [25,26,27] featuring quantitative literature analysis of MOFs are present in the literature, to the best of the author’s knowledge, none of them focused on wastewater treatment applications of MOFs. Wang et. al. [25] published the very first bibliometric analysis of MOFs, in which 17,312 articles, published from 1991 to 2014, were extracted from the Web of Science and were analyzed to find major research applications of MOFs. This study concluded that gas storage, adsorption, separation, catalysis, drug storage, and delivery are the most widely discussed applications of MOFs. In this study, MOF literature as a whole was considered for analysis, rather than focusing on a particular application of MOFs. In the same year, Ho et. al. [26] published a similar bibliometric analysis, in which publications were extracted from WoS, published from 1900 to 2014. In this article, MOF literature as a whole was considered, and the main focus was exploring the performance of authors, countries, and institutes. This study found China was a leading research hub, whereas the USA was the most impactful country. Ogawa [27] used citation network analysis to conduct a comparative analysis of zeolite and MOF applications in different fields of research.

**Table 1:** Wastewater Treatment Processes using MOF-Based Adsorbents.

Type of Pollutants	MOFs	Adsorption Capacity (mg/g)	Remarks	Reference
Basic Red 46	NH_2_-MIL-125(Ti)	1296	High reusability and stability over three cycles	[28]
Basic Blue 41	NH_2_-MIL-125(Ti)	1257	High reusability and stability over three cycles	[28]
Cephalexin	PCN-777	442.48	Suitable for wastewater consisting of traces of antibiotics	[29]
Ciprofloxacin (CIP)	Fe_3_O_4_/HKUST-1	538	Fast adsorption rate, which could remove all the pollutants within 30 min	[30]
	NPC-700	416.7	Good recyclability	[31]
	ZIF-67	471.7	Fast adsorption, due to the presence of hollow Co_3_S_4_	[32]
Congo Red (CR)	Fe-MIL-88NH_2_	167.5	Endothermic process to remove CR from wastewater	[33]
	[Ni_2_F_2_(4,4bipy)_2_ (H_2_O)_2_] (VO_3_)_2_ 8H_2_O	242.1	Significant factors: the amount of adsorbent, Congo red concentration, and temperature	[34]
Dimetridazole (DMZ)	MIL-53(Al)	467.3	The flexibility of MIL-53(Al)’s pores to enlarge as the concentration of DMZ increased, induced a high adsorption capacity	[35]
H_2_S	Cu-MOF	98–199	-	[36]
Hg^2+^	[Cu_3_(BTC)_2_]*_n_*	714.29	Heavy metal removal	[37]
Melachite Green	MIL 100 (Fe)	146	-	[38]
	ZIF8@SiO_2_@ MnFe_2_O_4_	1010.2	Easy regeneration of magnetic adsorbent	[39]
Methyl Orange (MO)	Cr-BDC@MIL-101	132	As the pH of wastewater increases, the adsorption capacity of MIL-101 decreases; the large pore size of MIL-101 gave advantages to the adsorption capacity.	[40]
	UiO-66	454	Excellent at low pH	[41]
	ZIF-8@SiO_2_@ MnFe_2_O_4_	78.12	Easy regeneration of magnetic adsorbent	[39]
Methylene Blue (MB)	HKUST-1/GO	3.5	The concentration of MB produced a significant result in the adsorption capacity of HKUST-1/GO	[42]
	NH_2_-MIL-125 (Ti)	862	High reusability and stability over three cycles	[28]
	Amino-MIL-101(Al)	762	High adsorption, due to the good synergy between the amino group and cationic dye MB	[43]
	Co-Fe-LDH@UiO-66-NH_2_	555.62	High reusability, without any significant changes in the efficiency	[44]
Naproxen and Clofibric Acid	MIL-101	312	Fast adsorption compared to activated carbon, due to the large pore size of MIL-101	[45]
Minocycline (MC)	Fe_3_O_4_@ MIL-68 (Al)	248.05	The original structure of MIL-68 (Al) remains the same	[46]
Nitrobenzene	CAU-1	970	Easy regeneration	[47]
	MIL-68 (Al)	1130	Easy regeneration	[47]
Organophosphorus Pesticides	UiO-67 (Zr)	537,360	-	[48]
Oxytetracycline (OTC)	ED-MIL-101	325.7	Strongly pH-dependent	[49]
Rhodamine 6G	MgFe_2_O_4_@MOF	306.75	Fast adsorption within 5 min	[50]
Rhodamine B	MgFe_2_O_4_@MOF	219.78	Fast adsorption within 5 min	[50]
Rocephin	MIL-101-NH_2_ MIL-101	277.8 204.08	Higher adsorption due to the presence of amino groups	[51]
Tetracycline Hydrochloride (TC)	UiO-66-(OH)_2_/GO	37.96	Adsorption efficiency increased when the concentration of the solution was 20 mg/L	[52]

All of these mentioned studies, which conducted a bibliometric analysis of MOFs, have one common characteristic, in that the MOF literature was considered as a whole and no specific application of MOFs was discussed in detail. Recently, Liu [22] broke this trend by conducting a bibliometric analysis of MOF literature related to the environmental aspects of MOFs. It was the first focused bibliometric analysis of MOFs. Nevertheless, there is a research gap to conduct a focused bibliometric study of MOFs, to reveal recent advancements in wastewater treatment applications of MOFs. Therefore, the authors have taken the initiative to map this field, by conducting a bibliometric and content analysis.

## 2. Research Methodology

With advancements in technology, the available literature on a particular topic increases day by day, which motivates researchers to carry out quantitative impact-based research assessments, to understand the innovations in different fields. Bibliometric analysis is a quantitative analysis tool that has been widely used to map out research directions in different fields [53,54,55]. The methodology of conducting an effective bibliometric analysis can be summarized in three steps: (i) database selection, (ii) search criteria and data refining, and (iii) data analysis. 

In a bibliometric analysis, to obtain a greater accuracy, it is necessary to choose a suitable database. Nowadays, various databases are used to carry out bibliometric analysis, such as PubMed, Scopus, Web of Science (WoS), and Google Scholar [24]. PubMed contains data related to medicine and biomedical sciences, whereas Scopus, WoS, and Google Scholar provide data related to most scientific fields [24]. Scopus provides data for the period 1966 to the present, whereas WoS is one of the oldest databases and covers data for the period 1900 to the present. As for Google Scholar, the relevant data is not summarized anywhere [56]. The current study used the WoS database. WoS is the first broad-scope international bibliographic database and was previously known as Web of Knowledge and established by Eugene Garfield during the 1960s; and then later, in 1992, the Thompson Reuters company acquired it and changed its name to WoS. Since 2016, WoS has belonged to the Clarivate Company [57,58]. It is used for the selection of journals, evaluation of research articles, bibliometric analysis, and other tasks. WoS contains six main citation indexes: the Books Citation Index (BKCI), Social Sciences Citation Index (SSCI), Science Citation Index Expanded (SCIE), Conference Proceedings Citation Index (CPCI), Arts & Humanities Citation Index (A&HCI), and Emerging Sources Citation Index (ESCI). These all help in carrying out bibliometric analysis, and due to bibliometric analysis, the importance of the database has increased [59]. The selection of relevant high-quality literature is an important factor, which can impact the main conclusions obtained from a bibliometric analysis [60]. To ensure the reliability of the journal articles, the WoS database was used to search relevant articles. 

In database search criteria, the search terms used to find articles play a vital role. To retrieve articles related to MOFs, the search terms used were as follows:

(TS = ((“metal organic framework”) OR (“metal organic frameworks”) OR (“metal-organic framework”) OR (“metal-organic frameworks”) OR (“porous coordination polymer”) OR (“porous coordination polymers”) OR (“porous coordination network”) OR (“porous coordination networks”))) AND TS = (“wastewater *” OR “effluent *”)

The fact that different researchers have used these key terms to find articles related to MOFs testifies to their credibility [61,62]. A total of 1569 records appeared related to the search terms, published within the timeline of 2017–2021. After refining records to only those published in the English language and eliminating different types of book chapters, data papers, letters, and editorials, 1374 records were filtered out. To improve the accuracy of the bibliographic analysis, the above records were further refined by removing articles containing fewer relative data. A total of 1187 relevant articles were downloaded in *.txt format, with each file containing 1000 records, and they were proceeded for bibliometric analysis. 

**Figure 1:**
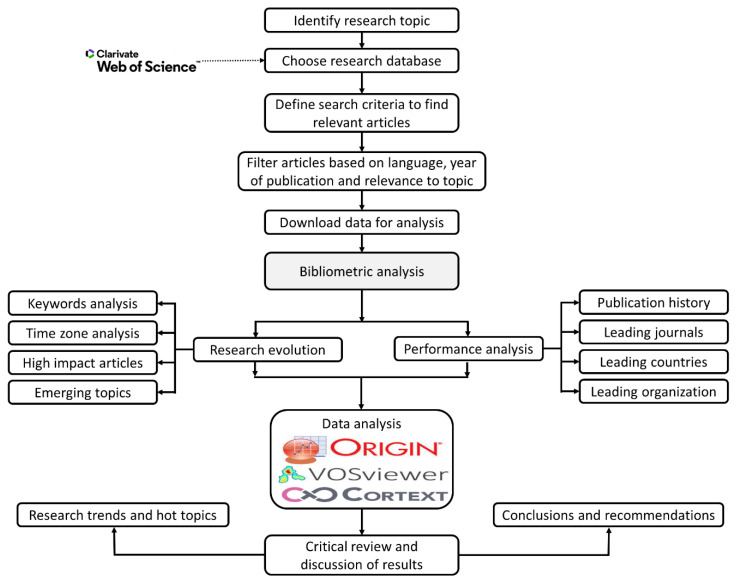
Research Methodology followed in the Study.

A bibliometric analysis analyzes a particular topic using different criteria, such as the performance of different countries, leading organizations, and authors; and also unearths recent research trends, the main journals for that topic, and their research themes. To conduct a bibliometric data analysis, VOS Viewer, CorText, and HistCite software were used. While performing this analysis, the collaborations between different authors and organizations was explored through a social network analysis. The social network analysis also analyzed different nodes and suggested the centralized and most influential nodes. The network analysis also highlighted the high frequency and co-occurrence of keywords, which enabled us to reveal the current hotspots and future research directions [18]. Furthermore, the intellective bases, emerging trends, and links between different research articles were obtained by using bibliometric coupling [63]. Figure 1 represents the detailed methodology used for conducting the bibliometric analysis.

## 3. Results and Discussion

On searching the core collection of WoS, a total of 1190 entities were found related to MOF for wastewater applications, during a span of 17 years from 2005 to 2021. A bibliometric analysis and a discussion of the results obtained were produced, as follows.

### 3.1. Publication Count

**Figure 2:**
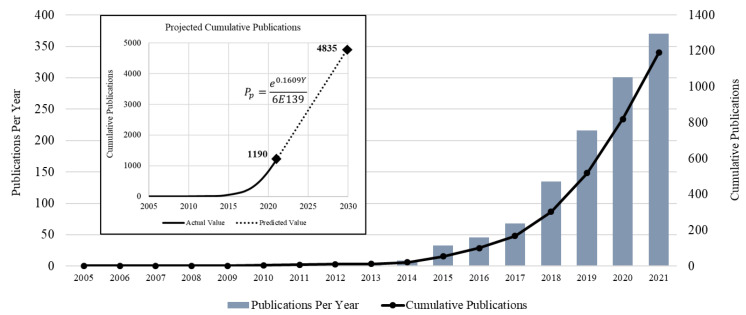
Research Growth in MOF for Wastewater Treatment Applications.

The total number of publications published related to MOF applications in wastewater treatment, and indexed in WoS, are shown in Figure 2. The authors searched the WoS database from 1970 to 2021; nevertheless, the first article revealed for MOF applications for wastewater treatment was found in 2005 [64]. The authors of [64] proposed a Cu-based MOF for pretreatment of olive oil mill wastewater (OOMW). Basically, the wastewater from these mills has a high content of polyphenols, which makes the wastewater nonbiodegradable and phytotoxic. Applying different chemical methods to treat it directly (without making it biodegradable) was not feasible, as post-processing or recovery of used chemicals was a major challenge. This drove the need to come with pretreatment methods capable of making OOMW a biodegradable product. The authors of [64] succeeded in this regard by employing a Cu-based MOF for OOMW, as a pretreatment option. In addition to significantly increasing the biodegradability, Cu-MOFs were observed to be capable of recovery and reuse after pretreatment of OOMW. On the other hand, the first patent [23] on MOFs was published in 1997, in which, for the first time, the author (Yaghi Omar M.) disclosed this class of crystalline solids, prepared by mixing a metal salt into an organic ligand in the presence of a templating agent. The inventors anticipated that these MOFs could find applications in wastewater treatment, specifically for decontaminating paper mill wastewater.

The number of articles published annually, showing the research growth in the subject under study, was plotted on a chart, along with the cumulative number of publications from 2005 to 2021, as shown in Figure 2. 

After performing a statistical analysis of the data of the publications, it was concluded that the cumulative number of publications showed a regular exponential trend, with the following equation:

(1) $$P_{p}=\frac{e^{0.1609Y}}{6\times 10^{139}}\;$$

where *P_p_* is the projected number of cumulative publications in a specific year *Y*. Using Equation (1), the total number of publications that are expected to be published by 2030 was estimated as 4835, which is more than four times the cumulative number of publications in 2021.

Overall, the past seventeen years of MOF application for wastewater treatment can be classified into two eras: an explorative era, and development era. The former refers to the time during which a topic is newly introduced and fundamental concepts are taking shape, whereas the latter refers to the era during which the topic matured and received attention from the scientific community. 

In the present study, the period between 2005 and 2013 was an explorative period, when the founding authors were working to build a conceptual framework for MOF applications in wastewater treatment. After the first publication in 2005 [64], discussing the application of Cu-MOF for OOMW, the topic remained off the radar until 2010, when the water stability of different MOFs was studied. Cychosz [65] broke the silence by employing the powder X-ray diffraction technique to study the water stability of MOFs and also studied the application of MIL-100 MOF for decontaminating pharmaceutical wastewater. It was observed in this study that stability is a function of a metal cluster of MOFs, where trinuclear chromium clusters were found to be most stable, followed by copper paddlewheel clusters and basic zinc acetate clusters. Discussing pharmaceutical wastewater treatment, the authors observed that MIL-100 is not only water stable but also has the capability to be a water pollutant absorbent (specifically furosemide and sulfasalazine), with large intakes at low concentration [65]. In 2012, a group of authors [66] presented another study using MOFs for the desulphurization of Hanford wastewater. It was observed that the metallic cation plays a vital role in determining the maximum concentration of pollutants that MOF can remove. By selecting an appropriate cation, crystallization can be controlled, to effectively reduce the concentration of pollutants. In 2013, a Chinese group of authors [67] reported a new MOF, Fe_3_O_4_@MIL-100, which was capable of removing polychlorinated biphenyls (PCBs) at trace levels from water samples collected from different rivers, lakes, and wastewater streamlines. PCBs were widely used between 1929 and 1997 as potential fluids in electrical insulating systems, capacitors, and transformers [68]. Although their production was banned in 1978, due to their highly toxic nature, their anthropogenic presence in natural waters and undocumented industrial applications remained an issue for decades after the ban. Therefore, it was pivotal to the research community at that time to reduce PCB concentrations in natural wasters, and this inspired authors to prove that MOFs were a promising sorbent in this regard. In the same year, 2013, another group of authors [69] opened a new path of research by synthesizing a large-pore mesoporous material capable of a tunable morphology, size, and porosity, by conversion of MOF, and reported the application of the same in decontaminating wastewater by removing methyl blue. This was the end of the explorative period and, afterward, MOF became a center of attention for the research community, and the subsequent years witnessed growing MOF applications in wastewater treatment. 

The development era of MOF applications in wastewater treatment started in 2014, and a steady growth in relevant literature was later observed. A wide range of research activities, spanning new techniques for synthesizing MOFs, introducing water repellent MOFs, and enhancing MOFs’ performance for wastewater treatment, were discussed in this era.

### 3.2. Citation Count

The most cited articles of a field provide information about the crucial concepts of that field. Analysis of these highly cited articles also highlights the timeline in which a particular idea originated [70]. Table 2 features the most cited articles, listed in descending order of total citations received, related to MOF applications for wastewater treatment. Of the top 10 most cited articles, five are original research articles, while five are review articles. The article ranked number one [71] is a review article that received the highest total citations, whereas the article ranked second [72] received the highest citations per year. Further analysis reveals that three out of 10 most cited articles were published by *The Journal of Hazardous Materials* which was identified as a high-focus and high-impact journal in this field. 

In [71], a detailed review study was conducted to summarize the degradation of organic pollutants using the photocatalytic activity based on MOFs. Review articles were divided into three portions, consisting of d-block metal MOFs, f-block MOFs, and polyoxometalate-based MOFs; and the recent progress related to all three sections was discussed. Surprisingly, two of the five most cited research articles shed light on Cr(IV) removal from wastewater.

### 3.3. Author Demography

The analysis revealed that 5071 authors have contributed to the study of applications of MOFs in wastewater treatment. About 76 authors have more than five publications and have received at least 50 citations. A list of the top ten most influential authors in this field is given in Table 3. The majority of the authors belong to China, followed by Iran and Saudi Arabia, which testifies to the findings of Table 4 and Table 5 as well.

### 3.4. Countries and Organizations

The different countries actively working on exploring MOF applications in wastewater treatment are compared in this section, based on both the quantity and quality of their research output. Quantity refers to the number of publications a country has, whereas quality refers to the number of citations received by the country. Such information is of particular interest for different organizations to collaborate and for job seekers to locate the market of interest.

**Figure 3:**
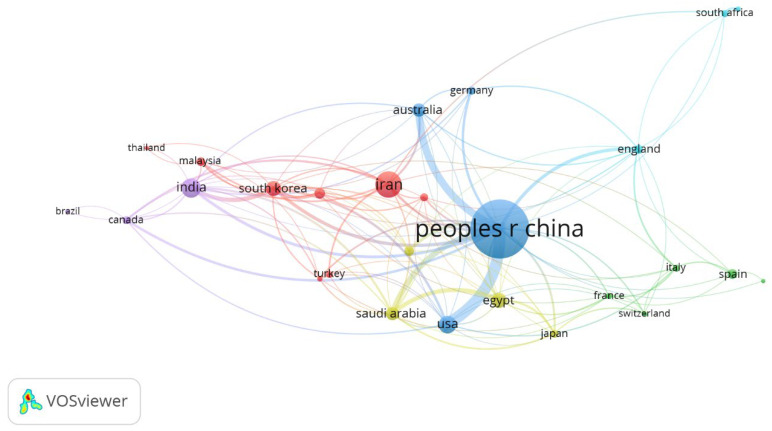
Leading Countries and their Collaborative Network in Research on MOF.

A total of 58 countries were found to be active in this research field, and only those having at least five publications to their credit were selected, filtering out 28 countries, as shown in Figure 3. The size of the circle represents the overall impact of a country, whereas the thickness of the line between two countries represents their collaboration network. China is the most prolific country in this field and has a strong collaboration network with Australia and the USA. Six clusters are observed in Figure 3. Cluster 1 (Red) is led by Iran, and has Malaysia, Pakistan, Singapore, South Korea, Thailand, Turkey, and Vietnam as members; cluster 2 (green) is led by Spain, and has France, Italy, Mexico, and Switzerland as members; cluster 3 (blue) is led by China, and has Australia, the USA, and Germany as members; cluster 4 (mustard) is led by Egypt, with Saudi Arabia, Japan, and Taiwan as members; cluster 5 (purple) is led by India, with Canada and Brazil as members; cluster 6 (aqua) is led by England, with South Arica and Nigeria as members. 

The top 10 influential countries are listed in Table 4. For the number of publications, China is the leading country followed by Iran, India, and the USA; whereas concerning the number of citations received, China is followed by the USA, Iran, and India. Countries were also compared based on their normalized contribution, calculated by dividing the number of articles by population. Singapore followed by Iran, Australia, and Saudi Arabia are the leaders in this category. 

A total of 1063 organizations were found to be active in this research field, and about 29 organizations have at least 10 publications to their credit. A list of the top 10 organizations is given in Table 5. Eight out of the top 10 organizations belong to China, which confirms the findings of Figure 3.

### 3.5. Journals and Disciplines

**Figure 4:**
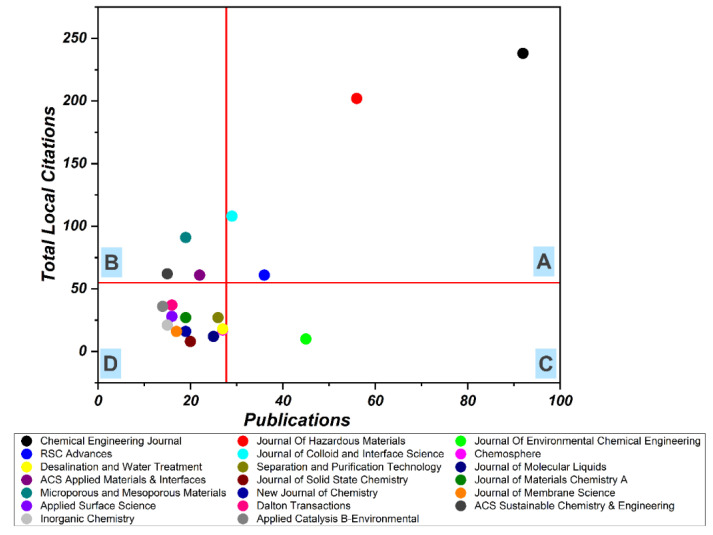
Leading Journals in MOF Research. Quadrant A denotes high focus and high impact journals, Quadrant B denotes low focus and high impact journals, Quadrant C denotes high focus and low impact journals, Quadrant D denotes low focus and low impact journals.

**Figure 5:**
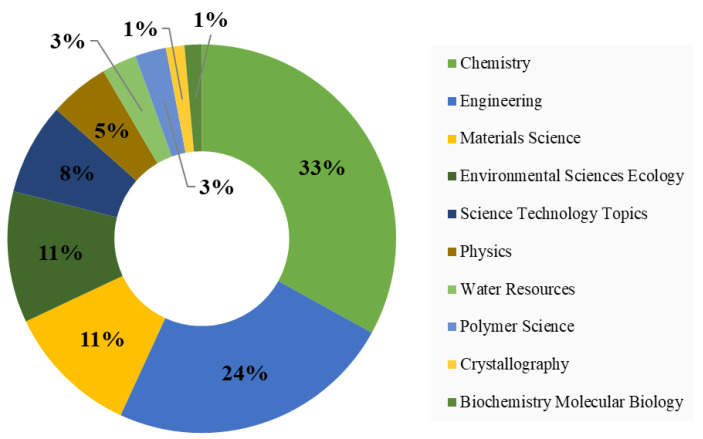
Major Research Disciplines in MOF for Wastewater Treatment.

The studies related to applications of MOFs for wastewater treatment were published in 260 journals, out of which 28 journals have published at least 10 relevant articles and received 100 citations. The top 10 journals in this field, see Table 6, accounted for 32.43% of the relevant literature. *Chemical Engineering Journal* is the leading journal in this field, in terms of both the number of articles published and citations received. The majority of the journals belong to the engineering, chemistry, and materials disciplines, which is in line with the findings in Figure 4 and Figure 5.

To quantify the impact of the different journals, the top 20 journals were selected and ranked using HistCite. Interestingly, the results of HistCite were the same as that of Table 2, except for the fact that using HistCite data, the journals could be categorized easily. Figure 4 represents the focus and impact of the top 20 journals in the field. The horizontal line in the graph depicts the average local citation received, whereas the vertical line represents the average number of articles published in a journal. These two lines divide the graph into four quadrants named A, B, C, and D. Quadrant A represents journals of high focus and high impact, and these are considered the most prestigious journals. This quadrant includes only four journals: *Chemical Engineering Journal*, *Journal of Hazardous Materials*, *RSC Advances*, and *Journal of Colloid and Interface Science*. Quadrant B denotes low focus and high impact journals and encircles only three: *ACS Applied Materials & Interfaces*, *Microporous and Mesoporous Materials*, and *ACS Sustainable Chemistry & Engineering*. Quadrant C denotes journals with a high focus and low impact, this category has only one journal: *The Journal of Environmental Chemical Engineering*. Quadrant D denotes journals that have low impact and low focus. The majority of the journals among the top 20 lie in quadrant D, which means studying MOFs applications in wastewater treatment is quite an interdisciplinary topic and a wide range of researchers affiliated with various research disciplines are exploring this field.

Figure 5 represents the top 10 disciplines active in MOF research for wastewater treatment. Chemistry is the dominant field that includes the synthesis of MOFs, controlling morphology, crystal structure, porosity, and many other relevant studies of MOFs. Furthermore, the performance enhancement of MOFs for wastewater treatment also comes under the shadow of this discipline. Engineering disciplines cover a broad spectrum of topics dealing with reactor design and optimization. The top disciplines depicted in Figure 5 are in line with the findings in Table 6.

### 3.6. Keyword Reoccurrence

This section sheds light on the research evolution of MOF applications in wastewater treatment and unearths intellectual structures, research trends, and emerging topics in this field. For this purpose, the keyword analysis technique was used. In this technique, the main keywords depicting the opinion of the authors are extracted from the literature, and based on these keywords, hot topics of the past and present are highlighted [81,82,83].

**Figure 6:**
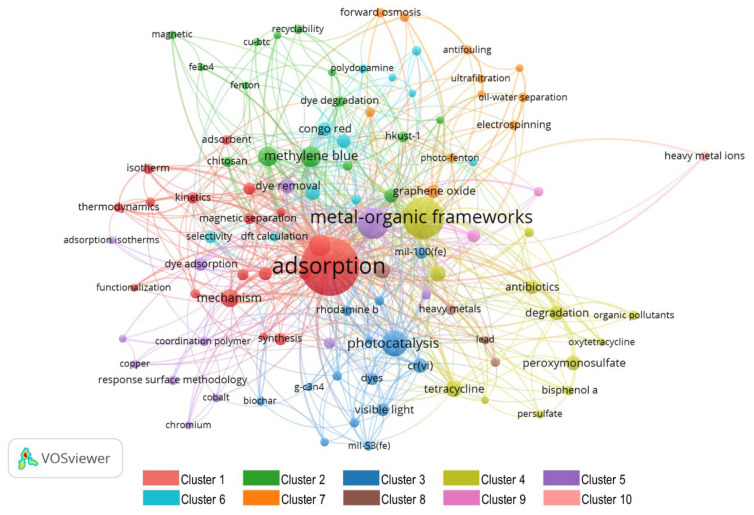
Most Frequently used Keywords in MOF Research.

A total of 2439 authors’ keywords were detected, out of which 80.19% appeared only twice, whereas only 4.75% (116) appeared more than five times and were selected for the analysis. These selected keywords, mapped in Figure 6, are frequently discussed topics related to the main research theme of MOF application for wastewater treatment.

In Figure 6, the size of the circle depicts the frequency of occurrence, which implies a circle with a large radius shows a keyword of high impact. The line joining two circles shows the relation of keywords at both ends. The thicker the line, the higher the co-occurrence of keywords. There are 10 colors in Figure 6, each denoting a different cluster.

Cluster 1 relates the adsorption characteristics of MOFs to wastewater treatment. Throughout the literature, adsorption is the most utilized application of MOFs that has been used for a wide range of purposes, including removal of harmful materials from liquid/gas phases, gas storage, separation, purification, catalysis, drug delivery, and so on [84]. The unique characteristics of MOFs, such as large surface, tunable pore size, and options for chemical functionalities (for selective adsorption) make them a promising candidate for adsorption; being low cost, low energy-intensive, less harmful, easy to operate, and having a simple design process [84,85]. Furthermore, this cluster reveals that understanding and enhancing the adoption capability of MOFs has been a topic of keen interest for researchers. For this purpose, thermodynamic modeling of adsorption mechanisms (adsorption enthalpies, adsorption entropies, and Henry’s adsorption constants) and applying compactional techniques such as DFT (density functional theory) were the topics of prime interest. Furthermore, the functionalization of MOFs has also played a vital role in the performance enhancement of MOFs. Although there is a wide range of MOFs being used for wastewater treatment, due to their exceptional absorptivity; UiO-66-NH_2_ and ZIF-8 were found to be the top choice of researchers. Both of these MOFs are well known for their exceptional adsorption capacity and high stability, which have encouraged researchers to exploit them in wastewater treatment [86,87]. 

Cluster 2 mainly represents the application of MOFs in dye degradation of industrial wastewater. Dyes are synthetic organo-complexes that are used as colorants, especially in textile industries [88]. During the dyeing process, the complete amount of dye is not utilized, and a substantial amount remains present in the wastewater. Direct release of this wastewater to water bodies causes environmental and health problems. Therefore, dye degradation, a process in which large and complex structures break down into small and simple structures with H_2_O and CO_2_ as by-products, is considered vital for decontamination of industrial water. Studies have revealed MOFs as an environmentally friendly, low cost, and a single-step process of dye degradation. The cluster analysis shows that degradation of methylene blue by adsorption, photocatalysis, and Fenton-like catalysis, using MOFs as the main agent, has been a favorite topic of discussion in the research community, as indicated by the high frequency of this keyword. Furthermore, applications of magnetic MOFs for organic dye removal were also widely discussed. Interestingly, observing recyclability, and the use of cellulose and chitosan for dye removal, shows the attention given toward environmentally-friendly and biodegradable MOFs.

Clusters 3, 4, and 5 relate to photocatalytic applications of MOFs for wastewater treatment, especially dye removal or dye degradation. Although there are many technologies available for pollutant degradation of industrial wastewater, most of them only separate pollutants and require additional postprocessing. Nevertheless, photocatalytic application of MOFs has gained attention as a promising method of pollutant destruction, due to their high efficiency, easy handling, and easy reproducibility [71]. Within photocatalytic applications, visible light-based photocatalysis was identified as the most widely discussed topic, because it is the best way to utilize the maximum solar potential [89]. Furthermore, using carbon-based materials such as graphene, biochar, and activated carbon has also been a topic of interest. Among the many optimization techniques, the response surface methodology was the most favored among authors. 

Cluster 6 correlates the selective adsorption of MOFs towards different pollutants such as methyl orange, p-nitrophenol, and 4-nitrophenol to DFT. Although adsorption is widely considered a low-cost process for dye removal [90], improper desorption of dyes may cause secondary pollution [91,92]. Therefore, selective adsorption emerged as a potential topic, and MOFs soon became a potential candidate, as MOF surface modification showed promising potential for selective dye removal [91]. To study surface modification, the computational technique DFT has been largely explored, which helps authors to simulate MOF structures, and computationally characterize and find optimal configurations of MOFs helpful for selective dye removal. 

Cluster 7 unearths the role of nanotechnology in enhancing the desired properties and characteristics of MOFs for their application in wastewater treatments. The role of graphene oxides and nanoparticles is very prominent among the other methods. Graphene oxides have been proven to be a potential precursor for making hybrid MOFs, because of their low cost and ease of functionalization [93,94]. Another advantage of using graphene oxides and nanoparticles is the increased adsorption capacity of MOFs, their easily tunable nature, and excellent physicochemical properties [94]. The most widely used techniques for nanostructured MOFs were identified as electrospinning, nanofiltration, and ultrafiltration. 

Cluster 8–10 sheds light on heavy metal removal from wastewater using MOFs. Due to some unique properties of MOFs, such as their high adsorption capacity, high selectivity, and excellent reusability [95], MOFs have gained attention recently. However, this application of MOFs is still in the development phase and faces numerous challenges in finding practical applications.

Overall, the top 15 most frequent keywords are listed in Table 7. Adsorption is the most frequent keyword, as MOFs were identified as potential candidates for wastewater treatment due to their exceptional characteristics. Photocatalysis is the second most frequent wastewater treatment application of MOFs. Some researchers have even preferred it over adsorption, because in photocatalytic degradation, pollutant is completely removed and no post-processing is needed, as in the case of adsorption. The main application of MOFs in wastewater treatment is dye removal, especially from textile industry wastewater. Furthermore, to enhance the absorptivity and performance of MOFs, graphene oxides have been identified as a favorite option.

### 3.7. Time Zone Analysis

**Figure 7:**
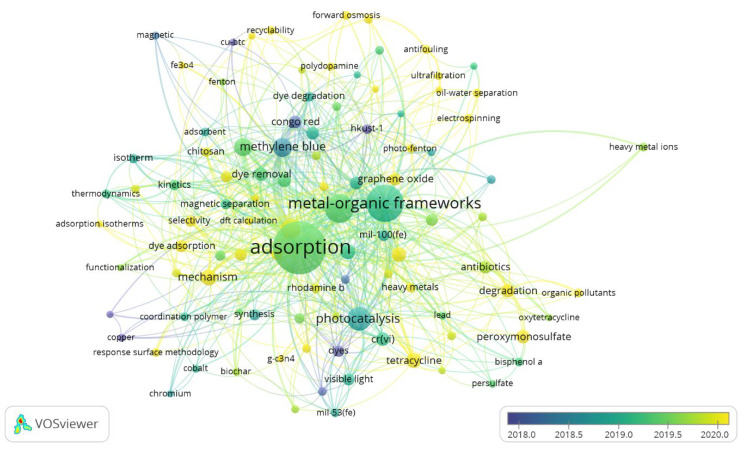
Time Zone Analysis of Research on MOF for Wastewater Treatment.

It is of prime importance to know how different topics emerged or evolved. This helps researchers to map the research field and identify topics of prime interest. Figure 7 depicts a time zone analysis of the MOF applications in wastewater treatment. Although data spanning the last 50 years was used, most of the frequent topics related to wastewater treatment emerged in the last 5 years, during 2017–2021. Initially, most of the research discussion was related to MOF’s porosity and its application to remove dyes from textile water. Removal of Congo red dye was the main focus in the initial period. To enhance performance, graphene played a vital role. 

Later on, computational modeling became fashionable and much work was done related to kinetic studies and thermodynamic modeling of MOFs. Furthermore, MOFs found further applications in oil–water splitting and heavy metal removal. Most recently, performance enhancement using nano-based techniques, such as nanoparticles, graphene oxide, ultrafiltration, and nanofiltration, have been observed as hot topics.

## 4. Emerging Research Trends in the Use of MOF for Wastewater Treatment

Emerging research fronts are those topics that have received substantial interest in recent years. To spot such topics, research articles published during the last three years were analyzed using keyword analysis.

The use of MOFs as a potential candidate to remove antibiotics, especially tetracycline from wastewaters is an emerging topic. Different antibiotics are used by a wide range of industries, the dairy industry and aquaculture specifically, because of their low cost, prompt effects, and wide-ranging treatment spectrum [96]. However, substantial amounts of such antibiotics are released into wastewater, which ultimately becomes part of the food chain. The presence of antibiotics in the food chain poses latent threats to the environment and humanity, in both the short term and long term. Due to the COVID-19 situation, the use of antibiotics has increased by many folds, which has made it a necessity to come up with ideas to remove antibiotics from wastewater [97]. Therefore, the attention toward the use of MOFs for antibiotics removal is not an exception. This is one of the recently emerged topics in the MOF field that needs to be explored. The performance enchantment of MOFs, in terms of a high adsorption capacity and water stability, was identified as an emerging topic. To cater to these issues, the application of graphene [98], graphene oxides [99], nanoparticles [100], and quantum dots [101] was also observed on various research fronts.

## 5. Key Challenges Faced by MOF Application in Wastewater Treatment 

Metal-organic frameworks (MOFs) have gained immense popularity in the past few years, owing to their ability to trap specific gases or liquids with high selectivity and recoverability. Their small size, high surface area, and highly porous volume makes them promising candidates for application in wastewater treatment. They also have the ability to remove volatile organic compounds (VOCs) and other pollutants from water, which makes them a great choice as an adsorbent or catalyst. With the increasing awareness about green chemistry and eco-friendly practices, there is a surge in demand for MOFs that can be used for removing pollutants from water. However, there are certain challenges that need to be overcome before they can be effectively applied in wastewater treatment. Some of the most important challenges faced by MOFs for this purpose are as follows:

### 5.1. Lack of Standardization and Quality Control

Metal-organic frameworks (MOFs) are a novel class of materials that have been attracting attention due to their extraordinary potential for wastewater treatment. However, there is a lack of standardization and quality control in the production and use of MOFs for this application, which has impeded their widespread adoption. This has led to the poor performance of MOFs in wastewater treatment applications, culminating in costly catalysts, increased sludge accumulation, and the fouling of biological treatment systems. To address this problem, it is important to understand the mechanisms by which MOFs degrade pollutants and the quality control measures that need to be implemented to ensure consistent performance. It is also important to develop standardized building blocks and conceptually design MOFs to facilitate reproducibility and quality control in manufacturing. These efforts will require concerted efforts from foundations, industry, and academia. In the meantime, the current lack of quality control and standardization of MOFs in wastewater treatment systems will continue to impede their wide-scale deployment.

### 5.2. High Cost of Operation

Another challenge faced by MOFs for application in wastewater treatment is their high cost of operation compared to other adsorbents or catalysts used in wastewater treatment. The cost of operation depends on various factors, such as the cost of raw materials, the quantity required, and the process used for the treatment. Depending on the type of MOF used, the cost of operation can vary. For example, zeolitic imidazolate frameworks (ZIFs) are relatively cheaper than metal-organic frameworks (MOFs), due to their lower binding capacity. However, MOFs are superior to ZIFs in terms of selectivity, which makes them an ideal choice for this purpose. This, in turn, makes the operation cost higher.

Another challenge faced by MOFs for application in wastewater treatment is their limited adsorption capacity. In comparison to other adsorbents or catalysts used in wastewater treatment, MOFs have a low binding capacity, owing to their large pores. As a result, large quantities of MOFs will be required to remove a small amount of pollutants from water. The large pores of MOFs make them a good choice for VOC removal. However, for the removal of other large molecules such as proteins and DNA, a large quantity of MOFs will be required. As a result, the cost of operations will go up.

### 5.3. Difficulties in Scaling Up the Process and Storage Issues

MOFs are widely used in the synthesis of sensors, membranes, and actuators, due to their high adsorption capacity and selectivity. However, they face a few difficulties when being applied in wastewater treatment, especially in terms of scaling up the process and storage of MOFs. This is mainly due to the fact that MOFs are synthesized in a batch process and are not produced in a continuous mode. As a result, they are not commercially available at large scale. Furthermore, MOFs are synthesized using a mixture of metal salts and organic linkers. As a result, they cannot be stored for long. This makes the process of scaling up the process of wastewater treatment difficult.

### 5.4. Lack of Durability

Another challenge faced by MOFs for application in wastewater treatment is a lack of durability. MOFs are synthesized using metal salts and organic linkers. As a result, they are not durable and are prone to corrosion. This makes them unsuitable for application in wastewater treatment, where the pH and other parameters are constantly changing. MOFs are also sensitive to light, heat, and pressure, which can lead to deactivation in a short period of time. If a MOF deactivates, the pollutants cannot be removed from the water and will remain trapped inside the MOF.

### 5.5. Limited Availability of Raw Materials for the Production of MOFs

The availability of raw materials for the production of MOFs is another challenge faced for application in wastewater treatment. MOFs are synthesized using metal salts and organic linkers. As a result, they are not sustainable and do not provide any added value to the environment. The majority of metals used in the synthesis of MOFs are rare, which makes them a challenge for large-scale production. In addition, organic linkers are also limited, which makes the production of MOFs uneconomical. The need for a sustainable and eco-friendly solutions for wastewater treatment has led to the search for alternative materials for the synthesis of MOFs. Certain metal oxides, such as ZnO, are being tested and are expected to be commercially available soon.

## 6. Conclusions

Based on the data extracted from the Web of Science related to MOF applications for wastewater treatment, a bibliometric and content analysis was conducted, to highlight the publication characteristics and research evolution of the field. The results show that the very first article in this field was published in 2005, in which a Cu based MOF was employed to decontaminate the wastewater of an olive oil mill. In recent years, a steady growth in literature has been observed; the last five years contributed 91.82% to the total publications available on this topic. A total of 260 journals were identified publishing research related to this topic, out of which the *Chemical Engineering Journal* was determined as the most prolific journal. Out of 58 countries active in this research field, China is the main research hub, with the highest number of articles published and citations received. Concerning the normalized contribution (articles/population), Singapore was the leading country. 

To unearth the intellectual structure of research streamlines, a content analysis technique was adopted. A total of 2439 keywords were detected, out of which only 4.75% appeared more than five times and were analyzed to highlight the research evolution. Overall, the adsorption and photocatalytic applications of MOFs to decontaminate wastewater were the most frequently discussed topics. Among a wide range of MOFs reported in the literature, ZIF and UiO-66 appeared among the frequent keywords. For MOF applications, dye removal was the most explored. 

A time zone analysis of research keywords revealed that, in the initial days, the main focus of researchers was using MOFs for dye removal from industrial wastewater, and later on attention shifted toward computational modeling, and thermodynamic and kinetic analysis of MOFs. After that, the performance enhancement of MOFs became popular, and the use of nanoparticles, graphene, and graphene oxides emerged as hot topics. Presently, the use of MOFs for heavy metal and antibiotic removal are emerging topics in this field, which need to be explored in the days to come. A major issue facing these applications is the low performance and low stability of MOFs in solutions. To resolve this, quantum dots, nanoparticles, and graphene oxide were identified as emerging research fronts.

## Figures and Tables

**Table 2:** Most Cited Articles Related to MOF.

Rank	Title	Ref. No.
1	Photocatalytic organic pollutants degradation in metal-organic frameworks	[71]
2	Highly Stable Zr(IV)-Based Metal-Organic Frameworks for the Detection and Removal of Antibiotics and Organic Explosives in Water	[72]
3	Adsorptive removal of hazardous materials using metal-organic frameworks (MOFs): A review	[73]
4	Cooperative insertion of CO_2_ in diamine-appended metal-organic frameworks	[74]
5	Doping of graphitic carbon nitride for photocatalysis: A review	[75]
6	Facile synthesis of amino-functionalized titanium metal-organic frameworks and their superior visible-light photocatalytic activity for Cr(VI) reduction	[76]
7	Towards the use of metal-organic frameworks for water reuse: a review of the recent advances in the field of organic pollutants removal and degradation and the next steps in the field	[77]
8	MIL-53(Fe) as a highly efficient bifunctional photocatalyst for the simultaneous reduction of Cr(VI) and oxidation of dyes	[78]
9	Removal of emerging contaminants from the environment by adsorption	[79]
10	Recent advances for dyes removal using novel adsorbents: A review	[80]

**Table 3:** Leading Authors of Research on MOF for Wastewater Treatment.

Authors	Publications	Citations	Organizations	Country	Research Area
Mahmoodi, Niyaz Mohammad	16	924	Institute for Color Science and Technology	Iran	Chemistry, Engineering, Wastewater Treatment
Wang, Peng	12	376	Shandong University	China	Chemistry, Engineering, Crystallography
Wang, Shixing	12	256	Nanjing University	China	Engineering, Environmental Ecology, Chemistry
Cao, Jiao	11	515	Hunan University	China	Chemistry, Engineering, Material Science
Huang, Hongliang	10	352	Tiangong University	China	Chemistry, Engineering, Material Science
Wang, Chen	10	127	Kunming Univ of Science and Technology	China	Chemistry, Engineering, Material Science
Xiong, Weiping	10	331	Hunan University	China	Chemistry, Engineering, Material Science
Hayat, Tasawar	9	630	King Abdulaziz University	Saudi Arabia	Engineering, Chemistry, Environmental Ecology
Wang, Chong-Chen	9	352	Beijing University of Chemical Technology	China	Engineering, Environmental Ecology, Chemistry
Wang, Yan	9	181	Hunan University	China	Engineering, Environmental Ecology, Chemistry

**Table 4:** Leading Countries in Research on MOF for Wastewater Treatment.

Rank	Country	Publication Contribution%	Citation Contribution%	Normalized Contribution
1	China	61.16	48.04	0.50
2	Iran	12.38	6.57	1.75
3	India	6.57	4.10	0.06
4	USA	5.81	11.21	0.21
5	South Korea	4.13	2.97	0.96
6	Egypt	4.04	2.46	0.47
7	Australia	3.20	3.19	1.49
8	Saudi Arabia	3.20	3.11	1.09
9	Vietnam	2.11	0.64	0.26
10	Spain	1.77	0.93	0.45

**Table 5:** Leading Organizations in Research on MOF for Wastewater Treatment.

Rank	Organization	Country	Publication Contribution%	Citation Contribution%
1	Chinese Academy of Sciences	China	5.14	18.81
2	Hunan Univ.	China	2.78	11.95
3	Islamic Azad Univ.	Iran	2.70	1.97
4	Beijing Univ Chem. Tech.	China	1.68	5.34
5	Kunming Univ Sci. & Tech.	China	1.68	2.60
6	Soochow Univ	China	1.68	6.26
7	Nanjing Univ	China	1.52	3.48
8	South China Univ. Tech.	China	1.52	2.25
9	King Abdulaziz Univ.	Saudi Arabia	1.43	4.23
10	Tongji Univ.	China	1.43	3.23

**Table 6:** Leading Journals in Publishing Research on MOF for Wastewater Treatment.

Rank	Journal Name	Impact Factor	Document Contribution%	Citation Contribution%	Category
1	*Chemical Engineering Journal*	13.273	14.13	22.49	Engineering, Environmental Engineering, Chemical
2	*Journal of Hazardous Materials*	10.588	8.60	15.17	Engineering, Environmental Environmental Sciences
3	*Journal of Environmental Chemical Engineering*	5.909	6.91	2.56	Engineering, Environmental Engineering, Chemical
4	*RSC Advances*	3.361	5.53	4.6	Chemistry, Multidisciplinary
5	*Journal of Colloid and Interface Science*	8.128	4.46	5.5	Chemistry, Physical
6	*Chemosphere*	7.086	4.15	2.58	Environmental Sciences
7	*Desalination and Water Treatment*	1.254	4.15	0.51	Engineering, Chemical Water Resources
8	*Separation and Purification Technology*	7.312	3.99	2.81	Engineering, Chemical
9	*Journal of Molecular Liquids*	6.165	3.84	1.95	Chemistry, Physical Physics, Atomic, Molecular and Chemical
10	*ACS Applied Materials & Interfaces*	9.229	3.38	3.66	Materials Science, Multidisciplinary Nanoscience and Nanotechnology

**Table 7:** Most Frequently used Keywords in MOF Research.

Rank	Keywords	%Occurrence
1	Adsorption	17.731
2	Metal-Organic Frameworks	8.967
3	Wastewater Treatment	5.163
4	Photocatalysis	3.668
5	ZIF-8	2.446
6	Methylene Blue	2.310
7	UiO-66	2.106
8	Mechanism	1.698
9	Photocatalytic Degradation	1.291
10	Cr(VI)	1.155
11	Dye Removal	1.155
12	Peroxymonosulfate	1.155
13	Selective Adsorption	1.155
14	Antibiotics	1.087
15	Graphene Oxide	1.087

## Data Availability

The datasets generated and analyzed in the research are available from the corresponding author and can be furnished upon request.
